# A Framework for Extension Studies Using Real-World Data to Examine Long-Term Safety and Effectiveness

**DOI:** 10.1007/s43441-021-00322-8

**Published:** 2021-07-12

**Authors:** Mehmet Burcu, Cyntia B. Manzano-Salgado, Anne M. Butler, Jennifer B. Christian

**Affiliations:** 1 Department of Epidemiology, Merck & Co., Inc., Kenilworth, NJ USA; 2 Real-World Solutions, IQVIA, Durham, NC USA; 3 Washington University in St. Louis, St. Louis, MO USA

**Keywords:** Extension studies, Roll-over studies, Real-world evidence, Real-world data, Enriched studies, Observational follow-up, Long-term outcomes, Direct-to-patient, Patient-mediated data, Pragmatic approaches

## Abstract

Understanding the long-term benefits and risks of treatments, devices, and vaccines is critically important for individual- and population-level healthcare decision-making. Extension studies, or ‘roll-over studies,’ are studies that allow for patients participating in a parent clinical trial to ‘roll-over’ into a subsequent related study to continue to observe and measure long-term safety, tolerability, and/or effectiveness. These designs are not new and are often used as an approach to satisfy regulatory post-approval safety requirements. However, designs using traditional clinical trial infrastructure can be expensive and burdensome to conduct, particularly, when following patients for many years post trial completion. Given the increasing availability and access of real-world data (RWD) sources, direct-to-patient technologies, and novel real-world study designs, there are more cost-efficient approaches to conducting extension studies while assessing important long-term outcomes. Here, we describe various fit-for-purpose design options for extension studies, discuss related methodological considerations, and provide scientific and operational guidance on practices when planning to conduct an extension study using RWD. This manuscript is endorsed by the International Society for Pharmacoepidemiology (ISPE).

## Introduction

Understanding the long-term benefits and risks of treatments, devices, and vaccines is critically important for individual and population-level healthcare decision-making. How long a new product works and for whom, as well as what long-term risks are associated with the product, can be evaluated through long-term follow-up of patients exposed to a new treatment, vaccine, or device. Indeed, many safety concerns are either too rare or have a long latency period and may not be measurable in traditional clinical trials; therefore, extension studies, or ‘roll-over studies,’ allow for clinical trial participants to ‘roll-over’ into a second related study to continue to observe and measure long-term safety, tolerability, and/or effectiveness [1]. When designed and conducted well, extension studies can provide early evidence of long-term outcomes on a new drug, biologic, or device and may also provide access with no out-of-pocket cost to potentially beneficial products in development among patients who were taking them during the trial. Indeed, these designs are often used as an approach to satisfy regulatory post-approval requirements on safety, effectiveness, or duration of effectiveness (e.g., in vaccines).

Extension studies are not a new design—they have been around a long time. For example, an extension study was designed to follow patients who participated in the West of Scotland Coronary Prevention Study (WOSCOPS), which was the first clinical trial of pravastatin therapy in the primary prevention setting of hypercholesterolemia. In the WOSCOPS, over 6,500 males with elevated low-density lipoprotein cholesterol (LDL-C) were randomized, between February 1, 1989 and September 30, 1991, to treatment with pravastatin 40 mg or placebo [2]. Initial five-year follow-up within the trial found a 26% reduction in LDL-C and 31% reduction in nonfatal myocardial infarctions and cardiovascular death. An extension study that continued assessments at 10, 15, and 20 years post randomization continued to measure cardiovascular benefits despite the fact that statin use beyond the 5-year clinical trial period was only 35.2% of placebo and 38.7% of pravastatin treated patients. In another example, children who were randomized to receive treatments or placebo for 14 months in the Multimodal Treatment of Attention Deficit Hyperactivity Disorder (MTA) study in the early 1990s were invited to return to the MTA clinics every 1–2 years for further assessments. This prospective follow-up aimed to assess any long-term effects—6–8 years after randomization—of the 14-month treatments in the MTA study [3].

Longer term assessments of clinical effectiveness and safety, such as shown from the WOSCOPS extension or MTA study, address important clinical and public health questions and are often required for regulatory post-approval safety studies. However, these designs can be expensive to conduct, particularly, when following patients for 10 to 20 years post trial completion. For example, consider the current evidence needs of monitoring the long-term safety and effectiveness of COVID-19 vaccines that are currently being distributed on a global scale—the cost and human resources of continuing to follow patients in large scale trials would be cost prohibitive. Given the increasing availability and access to real-world data (RWD) sources, direct-to-patient (DtP) technologies, and novel real-world study designs [4, 5], there are more efficient approaches to conducting extension studies while assessing these important clinical questions. A recent use case is a real-world extension study of a phase III clinical trial of the quadrivalent human papillomavirus (qHPV) vaccine in trial participants from selected countries (Denmark, Iceland, Norway, and Sweden) [6]. With its real-world methodology, the study was able to successfully assess long-term effectiveness, immunogenicity, and safety for up to 14 years after the start of vaccination by harnessing real-world data from the Nordic national health registries [6]. Given the anticipated latency, the assessment of effectiveness of HPV vaccination on prevention of cervical pre-cancers and cancers usually requires more than 10 years of follow-up [6]. Conducting extension studies for > 10 years using a traditional clinical trial infrastructure with active patient follow-up (based on scheduled clinical visits by patients to trial sites) would be cost prohibitive. Using a unique Personal Identification Number (PIN), the RWD-based extension study was able to link between registries for comprehensive passive follow-up with near complete retrieval of registry data—as opposed to active, site-based follow-up requiring patient visits.

The growing capability of linking multiple RWD sources to provide a rich and full spectrum of clinical information will allow researchers to transform real-world health data collected for clinical care into meaningful evidence for future extension studies. This paper is intended to provide a framework to introduce various fit-for-purpose study design options and data capture models for extension studies, discuss related methodological considerations, and provide guidance on best practices when planning to conduct an observational, extension study. This manuscript is endorsed by the International Society for Pharmacoepidemiology (ISPE).

## Overview of Models for Extension Studies

As described in the WOSCOP example above, *traditional* extension study designs extend the follow-up after the completion of a randomized clinical trial (RCT) study for longer periods of time, albeit with fewer assessments and required visits, while maintaining reliance on trial sites and related resources including on-site clinical staff and investigators to engage with the study participants. With more practical approaches, real-world follow-up methods—that do not require the ‘traditional’ clinical trial infrastructure—can be used to reduce costs, minimize burden on sites, clinicians, and patients, and reduce data collection efforts with linkages to real-world healthcare data (e.g., with the use of HIPAA [Health Insurance Portability and Accountability Act]-complaint tokenization approaches) [7–9].

Prior to selecting a real-world vs. traditional clinical site-based approach to follow the participants upon completion of the clinical trial (often referred to as the ‘parent’ trial or original clinical trial that the individual enrolled in), it is important to consider the rationale for conducting the extension study, the timing of initiating the extension study in relation to when or if the parent trial has begun, the potential number of parent trials that may contribute patients to the extension study, the endpoints that will be captured, and the clinical and regulatory circumstances related to the extension study. Each of these factors will determine the approach for designing an extension study. Below, we present different approaches to designing an extension study, given various clinical and operational considerations.

**Figure 1.**
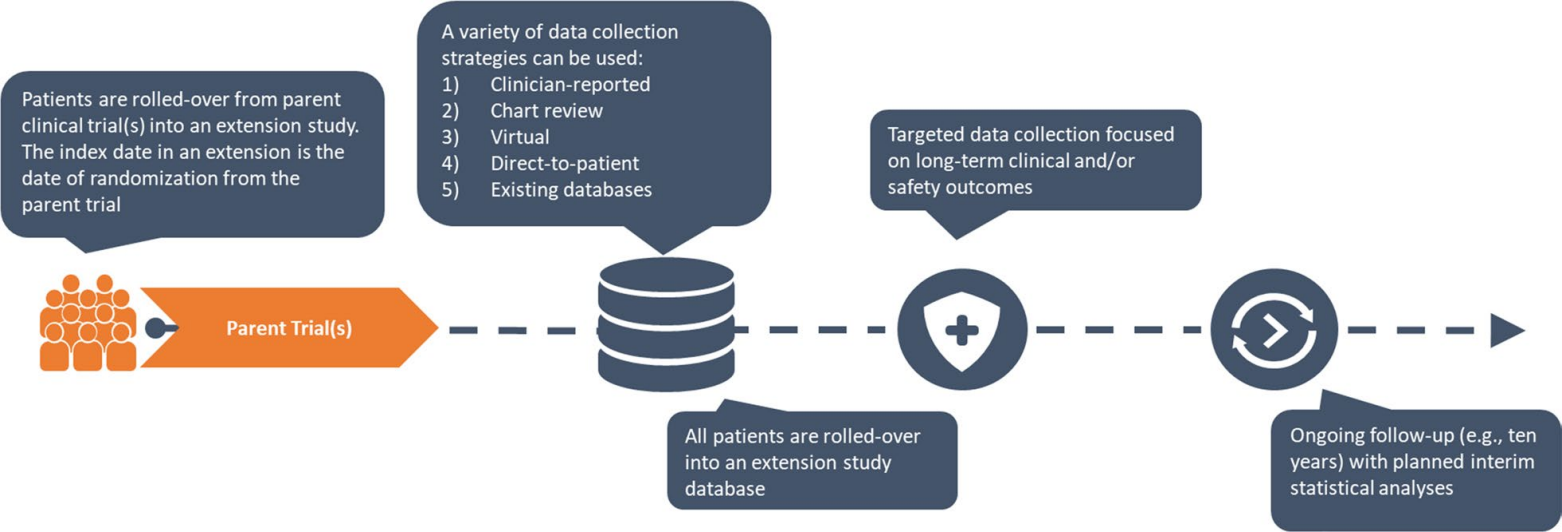
An Extension Study Model with Patients Rolled over from Parent Trial(s).

In extension study designs, the sites and patients targeted for participation are confined to those sites and patients that participated in the parent RCT, or nonrandomized single-arm trial. Extension studies with real-world follow-up are ideal when sites in the original parent trial can close and the extended follow-up can be observational and achieved, at least in part, via direct-to-patient registry design or through secondary uses of real-world data sources. In addition, no additional treatment is being provided and there are no endpoints requiring clinical assessments. Extension studies with real-world follow-up may also offer an efficient approach where several separate but related RCTs (e.g., multiple RCTs with extension needs for the same drug/device across several related indications) can be potentially rolled over into the same extension study. Figure 1 depicts a few potential models for extension studies, such as rolling over patients from one or more parent trials, a variety of data collection strategies, and variable follow-up times to consider. Depending on the country, there may be differences in the data collection strategies. Extensive experience with regional and local IRBs has shown that some countries allow direct-to-patient follow-up through advanced technologies while other countries mandate that clinicians and healthcare staff follow-up directly with patients. There are also circumstances where there may be several different arms, including arms that are continuing in a clinical trial infrastructure given the need to provide product and closely monitor safety until they can roll-over to an observational, real-world approach to follow-up.

In other circumstances, extension studies with traditional trial infrastructure may be preferable over an extension study with real-world follow-up. For example, at the end of the original parent trial, if the investigational drug is not authorized for marketing in the countries/regions where the trial was conducted, the real-world extension study design will not be feasible as the study will require storage and distribution of drug—that does not have marketing authorization—through clinical trial sites. Study drug, in most cases, would continue to be provided through the participating clinic sites. In other cases, regardless of marketing approval status of the drug, the extension of the ongoing parent trial with a clinical site-based follow-up approach may be preferred when numerous clinically complex/sophisticated endpoint constructs are to be collected, such as progression of autoimmune conditions (e.g., multiple sclerosis and/or rheumatoid arthritis) requiring imaging and clinical assessments.

In extension studies, the method of follow-up will depend on several factors and may vary by country or region due to local regulatory and/or ethics committee oversight rules and regulations. The different options of follow-up may include site-based approaches, the use of existing databases, direct-to-patient follow-up approaches including the use of technological advances and digital platforms/devices (e.g., smartphones and digital sensors), or hybrid patient follow-up/data collection approaches. With hybrid follow-up, an extension study can be designed to source data on the same patients through a mixture of multiple approaches, e.g., direct-to-patient approach for some endpoints (e.g., patient-reported outcomes/symptoms) enriched by the use of existing databases for other endpoints (e.g., hospitalization and/or emergency department visits through linkage to electronic health record/administrative claims data systems). Table 1 summarizes the key considerations of data capture approaches during follow-up of an extension “roll-over” study, such as the extent to which these approaches impact operational burden, patient burden, costs, and endpoint considerations.

**Table 1:** Key Considerations of Data Capture Approaches During Follow-Up of an Extension Study

	Data Capture Approaches				
	Clinician Reported	Chart Review	Virtual	Patient-Reported	Healthcare Claims and/or Electronic Medical Record Database
Site burden	High	Medium	Low	Low	Low
Patient burden	High	Low	Medium	Medium	Low
Cost	High	Medium	Medium	Low	Low
Endpoint considerations	– All endpoints – Most suitable for complex endpoints that may need original source of scans, imaging, invasive procedures, or clinical assessments	– Only endpoints that are captured during routine care – May need validation – May not be available in all regions/countries	– Endpoints that can be captured through virtual online connection (e.g., digital mobile devices)	– Endpoints that can be reliably reported by patients with no HCP* present – Validated PRO constructs may be used	– Only endpoints that are routinely captured and recorded in the database – May need validation – May not be available in all regions/countries

*HCP* Healthcare provider, *PRO* patient-reported outcomes.

## Methodological Considerations for Extension Study Designs

The first step before considering specific methodological design issues is to clearly define the research objectives of the extension study and to understand the stakeholder requirements for the study. Once these objectives and criteria are established, there are several important methodological considerations to include when designing extension studies: (1) define critical time points related to index date, follow-up times, exposure, and outcomes; (2) minimize selection bias into the extension study; (3) include a comparator to estimate long-term effectiveness and/or safety; (4) collect harder endpoints that are less subject to error; (5) minimize loss to follow-up and missing data; and (6) consider appropriate analytical approaches to further mitigate potential biases.

### Defining Critical Time Points

In an extension study, follow-up analyses of safety and/or effectiveness should consider several elements from the parent clinical trial. The index date, or time when patient follow-up begins, starts at the date of randomization of the patient into the parent study. For example, in an extension study in oncology, the time frame to evaluate overall survival should begin at the time from the index date (date of randomization) in the respective parent study to date of death due to any cause at the point of outcome assessment (which includes parent trial follow-up plus time during extension study follow-up). To do this type of analysis, trial data on select key variables such as date of randomization, treatment assignment, baseline demographics, and key trial outcomes of interest, must be linked to the extension study database for additional follow-up to ensure that all follow-up time since randomization is captured.

In most cases, time of randomization also aligns with date of treatment ascertainment. There are several approaches to assessing exposure. Most often, extension studies use an intention to treat approach, whereby patients who were randomized to the exposure of interest are categorized as such and patients randomized to placebo or an active comparator remain in that arm throughout the analysis period. However, it is important to capture changes in treatment over time, particularly, during the trial period but often during the extension period as well in order to conduct additional analyses that may assess time on treatment, discontinuation, switching, and the impact that these treatment changes may have on the outcomes of interest. Decisions around capture of exposure over time will depend on the route of administration of treatments, the purpose of the extension study, and the feasibility of capturing such information. For example, compared with an extension study examining long-term outcomes of an orally administered treatment, the extension study of an implantable device will be less subject to methodological issues related to adherence or changes in treatment during follow-up (due to the implanted nature of the treatment).

Outcomes of interest will be captured during the study period of the parent trial and then continued to be captured throughout the extension follow-up. For example, if major cardiovascular events such as hospitalization for myocardial infarction (MI) and death were assessed during the trial and these outcomes are continued to be evaluated in the extension study, then all events will count post index date (time of randomization) for time to event analyses and rate of outcomes. It will be important to link the data from the trial(s) with the extension follow-up so that all events are considered in the outcome assessment and time to first event will be captured across the entire follow-up period after randomization into the trial.

### Minimize Selection Bias and Enhance Recruitment Efforts

It is common for extension studies to only include patients that have a positive response to treatment in parent trials [10]. This can lead to selection bias and create a cohort of patients showing efficacy and tolerability to treatment by excluding patients that discontinued due to lack of efficacy or tolerability or that no longer want to continue with the treatment intervention. Providing information on the absolute number of responders enrolling into the extension study compared to the parent trials and a description of the responders and non-responders at the beginning of the extension study is recommended. In general, it is important to recruit as many patients as possible in extensions from the parent trial. A real-world extension study does not prohibit patients from initiating new therapies post trial completion, minimizing any ethical concerns among non-responder patients participating in observational follow-up since they may seek alternative treatments.

Several additional analyses are also recommended, including the assessment for the potential of selection bias. A main source of selection bias is that only patients who completed (and *survived*) the parent trials can enroll in the extension study [10]. To evaluate this selection bias, differences in demographic and baseline characteristics should be determined in patients who participate in the long-term follow-up from those who do not. If the reasons for patients not participating in the extension study are not independent of the treatment itself (e.g., lack of effectiveness, adverse event), then the denominator of response to treatment should include all patients that entered the initial parent trials. A worst-case scenario can be used as a sensitivity analysis, with the assumption that all patients that did not participate in the extension study are non-responsive to treatment [11].

### Include a Comparator Arm

To have meaningful interpretation of outcomes collected during an extension study, it is important to have a comparator arm in the extension studies. The selection of a comparator arm requires a complex array of considerations, including the route of administration of treatments, completion status of treatments from parent trials whether administration of treatments are completed [e.g., neoadjuvant systemic treatments preceding surgery], or needs to continue in the extension phase [e.g., anti-diabetic medications], regulatory status of treatments in the extension phase, and complexity of parent trials. For example, when more than one parent trial (with same experimental treatment but with different comparator arms) rolls into an extension study, complexities may arise in establishing a pooled comparator arm from these different but related parent trials. The heterogeneity of “control” treatments and potential differences in the patient population, timing, size, duration of individual parent trials need to be evaluated to establish a balanced and meaningful comparator arm. In some circumstances, it may not be feasible to roll patients from a comparator arm in a parent trial into the respective comparator arm of a subsequent extension study. Consider the randomized clinical trials of COVID-19 vaccines. Given the global public health emergency, in the post-approval setting, all patients globally ought to receive vaccines against COVID-19 as quickly as possible, unless there is a clinical reason otherwise (e.g., vaccine contraindication). In such cases, single-arm extensions focusing only on the treated arm of the parent randomized trials may be considered. However, in circumstances when control patients from the parent trials are not or cannot be rolled into an extension study, *external* control arms (including historical controls) can be established using real-world data to generate benchmark comparisons. Methodological and scientific considerations to generate fit-for-purpose external control arms, in general and for extension studies, are described in previous publications and are not within the scope of this paper [12–14].

### Select Endpoints that are Less Subjective to Error for Longer Follow-Up

One common issue related to observational follow-up post trial completion is that outcomes (such as hospitalization for myocardial infarction [MI]) may be measured differently in the trial compared to the extension period of follow-up. In the trial, a case report form with detailed data capture may be used to ensure that the hospitalization was due to the MI; however, observational follow-up (without required routine visits) relies on data that are recorded routinely as part of clinical care. For the MI hospitalization example, outcomes could be assessed through patient report, hospital or clinic charts, or billing records from health insurance claims and/or hospital electronic medical records. The date the event occurred will be recorded as the time of event. These data may not be structured or as detailed as the trial outcome information, but depending on the purpose can be suitable for capture of a major event and are unlikely to be differentially biased based on exposure status. Indeed, the other key point to consider is that the frequency of follow-up in extension studies is much less often than in a trial period (e.g., every 6 months or once a year), therefore, selecting endpoints that will be significant enough to recall by the patient and/or significant enough to seek medical care will be critically important to reduce missingness. For example, major cardiovascular events, reoccurrence or relapse of cancer, broken bones, major surgery, organ transplants or failure, and death are examples of harder endpoints that are significant and meaningful to measure over time. These harder endpoints are also less likely to be subject to information bias, due to recall or missingness, and suitable to measure from the patient (or next of kin) or from existing healthcare data sources, such as insurance claims and electronic medical records or registries. In certain circumstances, when existing RWD sources are chosen to assess more subjective endpoints, it will be necessary to conduct validation of real-world endpoints to ensure high specificity and sensitivity of operational algorithms used to define the endpoint of interest [15].

One mechanism to reduce information bias is to create a clinical outcomes adjudication or assessment committee, particularly, if more subjective endpoints are used or if the real-world definition differs from the trial outcome and a clinical review is needed. The role of a clinical outcomes assessment committee (COAC) is to provide a systematic, unbiased, and independent assessment of study outcomes using a set of predefined criteria (based on a COAC Standard Operating Procedure, or COAC-SOP) developed prior to the initiation of the extension study. In some circumstances, the parent trial may also need COAC, and in such cases, for consistency purposes, it will be important to utilize the same COAC both for the parent trial and the extension study, as feasible. The primary purpose of the COAC is to minimize bias and standardize the approach prior to initiation of the follow-up period. If possible, the COAC should review the outcomes of individual subjects without the knowledge of the exposure group in which the individual was assigned.

For international trials, it becomes even more challenging and important to define standardized outcomes that can be measured across health systems, languages, and countries, and may be captured by the patient, proxy, clinician, or existing data source. Use of a harder outcome—such as those described above—reduces the inherent variability introduced by these other factors.

### Minimize Loss to Follow-Up

Loss to follow-up (from parent trial to extension study and within the extension study) can be problematic and can compromise the validity of study findings [16]. The reasons for patient discontinuation are most likely *not* at random and the denominator for analysis should include all patients enrolled in the parent trials [11]. The impact of selection bias may be mitigated by performing an analysis on the intent to treat population as per the protocol in the parent trial [17], and as a sensitivity analysis, assume that any patient that discontinue the study is a non-responder in the extension trial (non-responder imputation). Sensitivity analyses using multiple imputation criteria for non-responders have also been proposed [18].

To minimize the number of patients that are loss to follow-up, the linkage of heterogenous data sources, depending on the country/region, may be considered for comprehensive follow-up data. For instance, in the United States, due to a fragmented healthcare system, patients often switch insurance coverage and may change the main point of healthcare. For example, one potential solution is to establish validated linkage approaches across various real-world data sources through privacy-preserving technologies, e.g., tokenization approaches (generation of anonymous identifiers that can be used to link patients’ data from various RWD sources). For survival-related outcomes, it is important to link available healthcare-based RWD sources with the National Death Index [19]. In other circumstances, to mitigate loss to follow-up within the extension study by reducing patient burden, a multitude of approaches can be used, including a hybrid approach of using available healthcare databases for follow-up combined with periodic follow-up by phone (e.g., annual check-in calls) or by other digital technologies to establish patient-centered health data sharing platforms [20].

### Analytical Considerations

In extension studies, estimation of the effects of treatment can be challenging for several reasons, including high dropout and/or loss to follow-up in the study population, especially over longer periods of follow-up. To minimize the effects of dropout and loss to follow-up, extension study protocols should simultaneously employ several analytic methods. These analytic approaches include an ITT analysis to estimate the effect of assigned treatment, as well as per-protocol and as-treated analyses to estimate the effect of received treatment. During protocol development, it is critical that researchers understand the advantages, disadvantages, and assumptions of each analytic approach. First, an ITT comparison is potentially challenging in settings in which a large proportion of participants have missing outcome data due to drop out or loss to follow-up [21]. In some cases, extension study analyses restrict the study population to those with complete data (i.e., complete case analysis). But, this approach is also potentially problematic because it assumes that the loss to follow-up occurs completely at random [11, 17]; and these effect estimates may be affected by selection bias in either direction [21]. While complete case analysis may be necessary in special circumstances, one recommendation is to try to determine the root causes of drop out or follow-up (to understand any potential biases introduced by complete case analysis), and increase targeted efforts to minimize loss to follow-up both proactively in modifying the follow-up tactics and reactionary such as through technologies, linkages to data, and direct-to-patient methods. The incorporation of real-world data linkages and direct-to-patient technologies can advance the methodological challenges experienced by traditional site-based extension studies with high dropout rates. While statistical methods are not within the scope of this paper, there are also methods to correct this potential bias using an appropriately adjusted as-treated analysis via inverse probability weighting, g-estimation, or instrumental variable (IV) estimation. An inverse probability weighted ITT analysis, for example, makes less assumptions where loss to follow-up occurs at random conditional on the measured covariates [21]. This is tempered, however, by the limitation that the validity of the adjustment relies on untestable assumptions about the unmeasured variables [22].

## Operational Considerations for Extension Studies: Timelines and Planning

Given the complex nature of extension studies, successful conduct and execution of these studies require significant operational support and concerted efforts in proactive planning with the “end in mind.” These include the following:

- Evaluation of inclusion of the extension study into initial “parent” trial protocol to maximize efficiencies
- Integration of the possibility of a need for an extension study into initial discussions with parent trial sites (if plans on an extension study are not finalized) or during the study conduct if considered at a later time
- Framing of informed consent to allow for easy transition to extension study (e.g., disclosure of longer-term data collection method/approaches and biospecimen storage)
- Inclusion and validation of patient-reported outcomes (PROs) or other patient-provided information (PPI) constructs in the initial “parent” clinical trial for later use in the extension study (as applicable)
- Validity and feasibility evaluation of utilizing tokenization approaches (generation of anonymous identifiers) to link “parent” trial patients with their records in various real-world data sources (e.g., administrative claims, electronic health records, genomic testing data, death, and other registries) [7]
- Possible incentives for patients (e.g., monetary, gift cards) for participation in additional follow-up beyond the clinical trial
- Possible reimbursement of services (treatment and clinical services) depending on the marketing authorization status and the requirements of the extension study
- Plans for possible flexible and hybrid models of site and staff involvement and related time and budget resource alignment with sites during the extension study, depending on the method of follow-up (e.g., real-world database follow-up only or hybrid approaches)
- Accounting for time on protocol development that can be implemented across different regions or sites depending on the method of follow-up. Because most registrational RCTs are global studies, most extension studies of these “parent” clinical trials are expected to cover the same regions, necessitating global harmonization and standardization of data collection methods in a timely manner.

While it is useful to assess these decisions prior to initiating the parent trial, more often, the need for an extension study may not become apparent until later during the conduct of the “parent” trial. For example, emergence of a new suspected adverse event during the conduct of the parent trial requires long-term follow-up, or regulatory circumstances to conduct an extension study to demonstrate long-term benefit:risk profile (e.g., long-term protection of vaccines, long-term survival data for a cancer treatment) is required. Regardless of the circumstances, the earlier that these operational considerations can be implemented, the more likely that the study participants can be consented prior to the parent trial ending. After the parent trial closure, it can be challenging to re-engage with the ‘parent’ trial participants.

## Conclusions

Given the increasing availability and access to RWD sources, direct-to-patient technologies, and novel real-world study designs, there are emerging efficient ways to conduct extension studies in the assessment of long-term outcomes of medical interventions. To date, there has been limited number of extension studies that use hybrid follow-up approaches with RWD and novel technologies. However, the world of development of medical interventions is rapidly evolving. The COVID-19 pandemic, its health system-related disruptions, and the urgency to develop evidence-based vaccines and medical interventions under emergency conditions are a reminder for the need for efficient and robust designs to conduct trials and collect high-quality long-term safety and effectiveness data from large patient populations [23, 24]. In this framework, we described various fit-for-purpose design options for extension studies that can incorporate non-traditional, real-world follow-up approaches and offered methodological considerations in the conduct of extension studies with RWE. Further methodological research and demonstration projects in the field of extension studies are needed to build consensus on RWD quality standards and metrics and on fit-for-purpose HIPAA-compliant patient follow-up and linkage tools in real-world settings.

## References

[1] Day RO, Williams KM (2007). Open-label extension studies: do they provide meaningful information on the safety of new drugs?. Drug Saf.

[2] Ford I, Murray H, Packard CJ (2007). Long-term follow-up of the West of Scotland Coronary Prevention Study. N Engl J Med.

[3] Molina BSG, Hinshaw SP, Swanson JM (2009). The MTA at 8 years: prospective follow-up of children treated for combined-type ADHD in a multisite study. J Am Acad Child Adolesc Psychiatry.

[4] Baumfeld Andre E, Reynolds R, Caubel P, Azoulay L, Dreyer NA (2020). Trial designs using real-world data: the changing landscape of the regulatory approval process. Pharmacoepidemiol Drug Saf.

[5] Sherman RE, Anderson SA, Dal Pan GJ (2016). Real-world evidence—what is it and what can it tell us?. N Engl J Med.

[6] Enerly E, Berger S, Kjær SK (2020). Use of real-world data for HPV vaccine trial follow-up in the Nordic region. Contemp Clin Trials.

[7] Agarwala V, Khozin S, Singal G (2018). Real-world evidence in support of precision medicine: clinico-genomic cancer data as a case study. Health Affairs (Millwood).

[8] Riordan HJ, Perakslis ED, Roosz S, Murphy M (2019). Utilising large data sets and extended trial observations to close the Alzheimer’s evidence gap. J Clin Stud.

[9] Zuidgeest MGP, Goetz I, Groenwold RHH, Irving E, van Thiel G, Grobbee DE (2017). Series: Pragmatic trials and real world evidence: paper 1. Introduction. J Clin Epidemiol.

[10] Taylor GJ, Wainwright P (2005). Open label extension studies: research or marketing?. BMJ.

[11] Hemming K, Hutton JL, Maguire MJ, Marson AG (2008). Open label extension studies and patient selection biases. J Eval Clin Pract.

[12] Burcu M, Dreyer NA, Franklin JM (2020). Real-world evidence to support regulatory decision-making for medicines: considerations for external control arms. Pharmacoepidemiol Drug Saf.

[13] Seeger JD, Davis KJ, Iannacone MR (2020). Methods for external control groups for single arm trials or long-term uncontrolled extensions to randomized clinical trials. Pharmacoepidemiol Drug Saf.

[14] Mack C, Christian J, Brinkley E, Warren EJ, Hall M, Dreyer N (2020). When context is hard to come by: external comparators and how to use them. Ther Innov Regul Sci.

[15] A Roadmap for Developing Study Endpoints in Real-World Settings. Duke Margolis Center for Health Policy. August 2020. https://healthpolicy.duke.edu/sites/default/files/2020-08/Real-World%20Endpoints.pdf. Accessed 27 Mar 2021.

[16] Greenland S (1977). Response and follow-up bias in cohort studies. Am J Epidemiol.

[17] Maguire MJ, Hemming K, Hutton JL, Marson AG (2008). Reporting and analysis of open-label extension studies of anti-epileptic drugs. Epilepsy Res.

[18] Buch MH, Aletaha D, Emery P, Smolen JS (2011). Reporting of long-term extension studies: lack of consistency calls for consensus. Ann Rheum Dis.

[19] National Death Index. National Center for Health Statistics. Centers for Disease Control and Prevention. https://www.cdc.gov/nchs/ndi/index.htm. Accessed 27 Mar 2021.

[20] Dhruva SS, Ross JS, Akar JG (2020). Aggregating multiple real-world data sources using a patient-centered health-data-sharing platform. NPJ Digit Med.

[21] Hernán MA, Hernández-Díaz S (2012). Beyond the intention-to-treat in comparative effectiveness research. Clin Trials.

[22] Toh S, Hernán MA. Causal inference from longitudinal studies with baseline randomization. Int J Biostat. 2008;4(1):Article 22. 10.2202/1557-4679.1117 PMC2835458 20231914

[23] Karzai F, Madan RA, Dahut WL (2020). The world of clinical trial development post COVID-19: lessons learned from a global pandemic. Clin Cancer Res.

[24] Annual Real World Evidence Conference: Applying Lessons Learned from RWE in the Time of COVID-19 to the Future. Duke Margolis Center for Health Policy. October 2020. https://healthpolicy.duke.edu/events/annual-real-world-evidence-conference-applying-lessons-learned-rwe-time-covid-19-future.

